# Evaluation of Knowledge, Attitudes, and Experiences of Perfusionists on the Safety of Cardiopulmonary Perfusion

**DOI:** 10.21470/1678-9741-2024-0340

**Published:** 2025-10-15

**Authors:** Turker Sahin, Ufuk Turan Kursat Korkmaz, Hakan Guven, Mustafa Selcuk Atasoy, Yusuf Velioglu, Ahmet Yuksel

**Affiliations:** 1 Department of Perfusion, Near East University Medical Faculty Hospital, Nicosia, Turkish Republic of Northern Cyprus; 2 Department of Cardiovascular Surgery, Faculty of Medicine, Bolu Abant Izzet Baysal University, Bolu, Turkiye; 3 Department of Cardiovascular Surgery, Bursa Medical Park Hospital, Mudanya University, Bursa, Turkiye; 4 Department of Cardiovascular Surgery, Bursa City Hospital, Bursa Faculty of Medicine, University of Health Sciences, Bursa, Turkiye

**Keywords:** Cardiopulmonary Bypass, Heart-Lung Machine, Perfusionist, Perfusion Safety, Perfusion Accidents.

## Abstract

**Introduction:**

Perfusion accidents still threaten the safety of perfusion during open-heart surgery. To prevent these accidents and increase perfusion safety, it is important to first determine the profile of perfusionists. The aims of this study were to determine the current status of perfusion safety during open-heart surgeries in Turkiye and to investigate the knowledge, attitudes, and experiences of perfusionists about cardiopulmonary perfusion safety.

**Methods:**

First, 148 perfusionists answered the Perfusionists’ Attitudes on Perfusion Safety survey, and second, 109 perfusionists answered the Perfusion Safety and Accidents survey. In the first survey, perfusionists’ attitudes and opinions help us understand the profile of Turkish perfusionists. In the second survey, we tried to obtain experiences and data about perfusion accidents, experienced by the participant perfusionists, to make a detailed analysis.

**Results:**

Turkish perfusionists are willing to ensure perfusion safety and prevent accidents. In this study, however, we found that educational institutions and clinics have not transmitted enough knowledge to perfusionists related to perfusion safety issues. There is some lack in the perfusionists knowledge about perfusion safety and preventing or reducing perfusion accidents.

**Conclusion:**

Perfusionists need more training to ensure perfusion safety and to prevent perfusion accidents. It would be very beneficial to include a course on perfusion safety in academic settings that focuses on this subject in the curricula. Further comprehensive studies on this subject will make very important contributions to the practical perfusion applications in increasing perfusion safety and reducing perfusion accidents.

## INTRODUCTION

**Table:** 

Abbreviations, Acronyms & Symbols	
ACT	= Activating clotting time
CPB	= Cardiopulmonary bypass
MiECC	= Minimally invasive extracorporeal circulation
PVC	= Polyvinyl chloride
RCTs	= Randomized controlled trials

Retrospective studies have demonstrated that at least 5% to 20% of patients presenting to the hospital encounter an adverse event^[1^,^2]^. Heart-lung (cardiopulmonary bypass [CPB]) machine, which is used as an extracorporeal system in open-heart surgery, and CPB applications performed with the component integrated to this machine pose a high risk for patient safety, and the risks caused by these applications can be life-threatening in some cases^[3]^. On the other hand, life-threatening mistakes may also occur during cardiovascular perfusion. It can be said that some of these mistakes result from the technologies used during the perfusion, while the others are due to the techniques used by the perfusionist and the surgeon.

Perfusionists are among the important members of a heart surgery team. The CBP system that they manage is the primary cause of perfusion-related accidents and undesired complications^[3]^. This system has a large surface that is foreign for body tissues and especially the blood. Being one of the most important components of open-heart surgery, the CPB system has been used since the first introduction of open-heart surgery and brings many complications and side effects^[4^,^5]^.

Perfusion safety is a multidirectional component that includes equipment and safety devices used during CPB, execution of the perfusion, surgical techniques used, the attention that must be paid, communication among the staff in the operating room, and even their educational status^[6]^.

Undoubtedly, each case carries a high risk for perfusionists and therefore it is inevitable for them to perfuse the patient in an uneasy and stressful way if adequate perfusion is not provided safely. This is very important, because perfusion accidents and complications are a serious threat to the patient and carry a high risk of death. For these reasons, increasing the perfusion quality by perfusionists is directly related to having systems and technologies that can prevent these accidents and complications^[7]^. Furthermore, having technology and systems that increase the safety of perfusion will decrease the perfusionist’s stress, reducing the risk of making mistakes.

The aims of this study were to determine the current status of perfusion safety during open-heart surgeries in Turkiye and to investigate the knowledge, attitudes, and experiences of perfusionists about cardiopulmonary perfusion safety.

## METHODS

### Study Population

Before the beginning of the study, ethical approval was obtained from the Karabuk University Non-Interventional Clinical Research Ethics Committee with the decision number 4/18, dated March 28, 2018. Additionally, the Turkish Society of Perfusionists was contacted. Contact information of all perfusionists registered in the society was obtained by the researchers. A member list was then created. Through the e-mails sent to the members, it was tried to reach 668 perfusionists who formed the universe of the study. The e-mail included an introduction of the researcher and a summary of the objectives of the research. The surveys were then prepared via Google forms, and the perfusionists determined were invited with e-mails to fill the surveys.

### Data Collection Tool

Data used in this study were collected through two surveys: the Perfusionists’ Attitudes on Perfusion Safety and the Perfusion Safety and Accidents. Questions used in these surveys were prepared by the researcher by screening the relevant current literature.

**Perfusionists’ Attitudes on Perfusion Safety**: This survey included five demographic questions and 63 further questions investigating opinions and attitudes of the perfusionists about cardiopulmonary perfusion safety. The questions were scored with 5-point Likert scale except for the five demographic questions. The answer options used in this survey included “strongly disagree”, “disagree”, “undecided”, “agree”, and “strongly agree”. Opinions and attitudes of the perfusionists were evaluated through five sub-scales in this survey. These sub-scales included 10 questions about written/registered perfusion safety tools, 11 questions about the use of consumable and disposable equipment, 15 questions about the use of safety equipment, 12 questions about perfusion safety and applications, and 15 questions about education of the perfusionists. This survey was answered by 148 perfusionists.

**Perfusion Safety and Accidents**: The survey consisted of 66 questions investigating opinions and experience of the perfusionists about the events and accidents that threaten perfusion safety.

### Statistical Analysis

Data obtained in this study were analyzed using the IBM SPSS Statistics for Windows, version 24.0 (IBM Corp., Armonk, N.Y., USA) software. The variables are expressed as percentage (%) and frequency (f) values.

## RESULTS

In this study, we applied two surveys: the Perfusionists’ Attitudes on Perfusion Safety with 63 questions and the Perfusion Safety and Accidents with 66 questions.

### Perfusionists’ Attitudes on Perfusion Safety

A total of 148 perfusionists participated in the first survey and answered the questions through the 5-point Likert scale. Of all participants, 100 (67.6%) were male, and 48 (32.4%) were female. A significant portion (44.6%) of the perfusionists were in the 40 - 49 years age group. Of all participants, 42.6% were graduates of perfusion associate degree programs , while 14.9% graduated from a license program such as nursing, physiotherapy, psychology etc.

Professional experience of the participants was found to be 16 years or longer in more than half (51.8%) of them. The rate of perfusionists who had the least (one - five years) experience was 14.8%. Of all participants, 40.3% (n = 70) stated that they perform ≥ 20 perfusion cases in a month. Responses of the participants were summarized and analyzed in three categories as “agree”, “undecided”, and “disagree”.

All participants agreed that a perfusionist should have a labour contract and that the perfusionist should use a checklist before each procedure. Opinions and attitudes of the perfusionists about perfusion safety tools are given in Table 1.

**Table 1:** Opinions of the perfusionists about perfusion safey tools.

Item	Agree	Undecided	Disagree
	n (%)	n (%)	n (%)
There should be a labour contract	140 (94.6)	4 (2.7)	4 (2.7)
There should be a job/task definition in the institution	148 (100)	0	0
Practice/training records related to perfusion should be kept and archived	148 (100)	0	0
There should be a written perfusion protocol in the hospital/operating room	148 (100)	0	0
Perfusion accidents/events should be recorded	144 (97.3)	4 (2.7)	0
Instructions of use/manuals for perfusion devices should be kept at hand	144 (97.3)	4 (2.7)	0
Records of routine perfusion equipment maintenance should be kept	148 (100)	0	0
A pre-perfusion checklist should be used	148 (100)	0	0
A post-perfusion checklist should be used	140 (94.6)	8 (5.4)	0
Electronic perfusion database program should be used	148 (100)	0	0

All of the perfusionists think that the heart-lung machine should have spares of important apparatus and catheters. Attitudes of the perfusionists towards the use of material are presented in Table 2.

**Table 2:** Attitudes of the perfusionists towards material usage.

Item	Agree	Undecided	Disagree
	n (%)	n (%)	n (%)
Expiry date of the consumables must be checked monthly	148 (100)	0	0
An arterial filter integrated or separate from the oxygenator must be used	143 (96.6)	5 (3.4)	0
Arterial and venous cannulas should not be resterilized	115 (77.7)	9 (6.1)	24 (16.2)
CPB system can be installed dry (without priming/dry-setup)	107 (72.3)	24 (16.2)	18 (11.5)
Prime solutions should be filtered using a pre-bypass filter	112 (75.7)	28 (18.9)	8 (5.4)
In elective cases, cannula, tubing, and oxygenator selection should be made one day in advance	139 (93.9)	9 (6.1)	0
Recording and reporting of consumables should be followed weekly	140 (94.6)	8 (5.4)	0
Plastic clamps (strips and tape gun) must be available close to the CPB system	148 (100)	0	0
A filter should be used in cardioplegia line	120 (81.1)	7 (4.7)	17 (11.5)
One-way valve should be used in aortic and intracardiac vent lines	141 (95.3)	7 (4.7)	0
The important apparatus of the whole set and spares of the catheters should be kept near the heart-lung machine	148 (100)	0	0

CPB=cardiopulmonary bypass

Opinions of the perfusionists about the use of equipment are shown in Table 3. All of the perfusionists reported that what the surgeon did during the operation should be strictly followed throughout the case. Perfusionists' opinions on perfusion safety practices are given in Table 4. Perfusionists' views on training, authority, and skills are given in Table 5.

**Table 3:** Attitudes of the perfusionists towards the equipment usage.

Item	Agree	Undecided	Disagree
	n (%)	n (%)	n (%)
An arterial line pressure transducer should be used	148 (100)	0	0
A cardioplegia line pressure transducer should be used	144 (97.3)	4 (2.7)	0
A venous line pressure transducer should be used	83 (56.1)	49 (33.1)	16 (10.8)
A mechanical manometer should be used for arterial line pressure	111 (75)	16 (10.8)	20 (13.5)
A flowmeter showing arterial blood flow should be used	136 (91.9)	12 (8.1)	0
A flowmeter to show cardioplegia blood flow should be used	107 (72.3)	33 (22.3)	8 (5.4)
In-line arterial blood gases should be monitored continuously	128 (86.5)	4 (2.7)	16 (10.8)
A blood level sensor should be used in the venous reservoir	148 (100)	0	0
Oxygen gas analyzer should be used	140 (94.6)	3 (2)	5 (3.4)
Oxygen saturation in venous blood should be monitored	140 (94.6)	4 (2.7)	4 (2.7)
Venous blood gases (pO2 and pCO2) should be monitored	124 (83.8)	16 (10.8)	8 (5.4)
Plasma lactate level in venous blood should be monitored	128 (86.5)	12 (8.1)	8 (5.4)
It is safer and more practical to control the venous line with a tubing clamp	77 (52)	33 (22.3)	38 (25.7)
It is safer to control the venous line with the electronic occluder	122 (82.4)	17 (11.5)	9 (6.1)
There should be a camera that sees the surgical field and a screen where the perfusionist watches and follows the images instantly	140 (94.6)	8 (5.4)	0

**Table 4:** Perfusionists' opinions on perfusion safety practices.

Item	Agree	Undecided	Disagree
	n (%)	n (%)	n (%)
The perfusionist should visit the patient before the case	103 (69.6)	29 (19.6)	16 (10.8)
The perfusionist should review the patient's file the day before the case and take the necessary notes	143 (96.6)	5 (3.4)	0
The perfusionist must see what the surgeon is doing in the surgery throughout the case	148 (100)	0	0
A second perfusionist should be working with during the case	143 (96.6)	5 (3.4)	0
Roller pump occlusion settings should be checked before each case	148 (100)	0	0
The heater-cooler water should be changed monthly	140 (94.6)	8 (5.4)	0
Samples should be taken for microbiological analysis during the heater-cooler water change	128 (86.5)	20 (13.5)	0
ACT value should be measured every half hour	136 (91.9)	3 (2)	8 (5.4)
If in-line blood gas monitoring is not performed, arterial blood gas should be checked every half hour	148 (100)	0	0
Spare oxygen gas cylinders should be available in the operating room	148 (100)	0	0
The perfusionist must have a flashlight throughout the case	107 (72.3)	12 (8.1)	30 (20.3)
The perfusionist must have a hand-crank with during the case	148 (100)	0	0

ACT=activated clotting time.

**Table 5:** Perfusionists' views on training, authority, and skills.

Item	Agree	Undecided	Disagree
	n (%)	n (%)	n (%)
In perfusion training, perfusion should be performed on the simulated patient before the real patient	148 (100)	0	0
Perfusionist candidate should be able to graduate from school only after gaining at least 50 - 100 case experience under the supervision of a supervisor perfusionist	145 (98)	3 (2)	0
Perfusionists must take the Qualification Exam (theoretical and practical exams) after graduation	148 (100)	0	0
Those who pass the Qualification Exam must not hold an indefinite certification	127 (85.8)	4 (2.7)	16 (10.8)
Recertification should be done to keep the knowledge of perfusionists up-to-date and alive	127 (85.8)	21 (14.2)	0
I know what to do if the patient develops malignant hyperthermia during perfusion	143 (96.6)	5 (3.4)	0
I know what to do in case of arterial line rupture/dislocation during perfusion and I can apply it	148 (100)	0	0
I know what to do if the arterial roller pump module fails during perfusion and I can apply it	148 (100)	0	0
I know what to do when the heater-cooler device breaks down and I can apply it	139 (93.9)	9 (6.1)	0
I know what to do if massive air enters the arterial line and I can apply it	148 (100)	0	0
I know what to do in case of an air-block as a result of massive air inflow in the venous line and I can apply it	148 (100)	0	0
I know what to do and can apply if the arterial filter breaks or leaks	148 (100)	0	0
I know what to do in case of massive clot formation in the venous reservoir and I can apply it	148 (100)	0	0
I know what to do in the event of a malfunction in the O2/gas mixer and I can apply it	144 (97.3)	4 (2.4)	0
I know when to change the oxygenator during the case and I can apply it	143 (96.6)	5 (3.4)	0
I know what to do if the electricity supply and batteries are completely cut/deplete and I can implement it	148 (100)	0	0

### Perfusion Safety and Accidents

This survey was conducted in order to evaluate knowledge and experience of perfusionists about cardiopulmonary perfusion safety. A total of 109 perfusionists responded to the survey, which consisted of 66 questions. However, due to space limitations, important points will be emphasized. The majority of the perfusionists stated that they had been educated on perfusion safety mainly through schools, seminars, symposiums, and congresses.

**Fig. 1:**
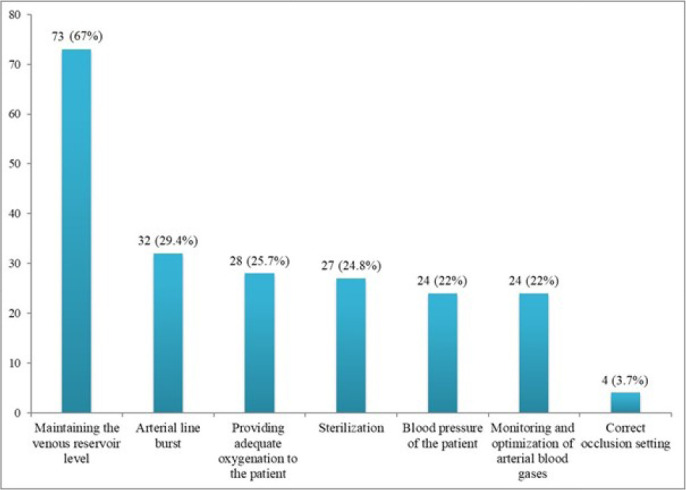
Perfusion safety perceptions of the perfusionists.

Responses of the participants regarding the perception of perfusion safety are shown in Figure 1. In this question, the perfusionists could mark more than one option. Ninety-two (84.4%) perfusionists marked all options. Seventy-three (67%) perfusionists marked the option of “protecting the venous reservoir level” as the most marked option.

Of the participants, 52.3% stated that they encountered a bubble problem between the oxygenator and the arterial filter in the CPB system. The rate of perfusionists reporting that they could perform the necessary intervention when this occurred was 67%.

Development of embolism was defined by 40.4% as the most important perfusion safety problem, 96.3% of the participants reported that they had to establish the CPB system in an emergency setting, and 40.4% of perfusionists encountered dissection at the arterial cannulation site during perfusion.

Forty-four percent of the participants encountered a monitor or screen failure in the heart-lung machine, 63.3% encountered oxygenator failure, and 73.4% had to change the oxygenator. Thirty-three percent of the perfusionists reported mechanical failure, and 31.2% an electrical/software failure while using the autotransfusion device. While 54% of the perfusionists reported a mechanical failure in the heater-cooler, and 51.4% an electrical failure, 67% reported that they encountered a mechanical/electrical failure in the activating clotting time (ACT) device.

Of the participants, 51.4% encountered mechanical failure in the arterial pump module, and 74.3% in the heart-lung machine; 33% answered “yes” to the question of whether the arterial cannula dislocated from the aorta during the case.

Forty-four percent of perfusionists reported that they encountered the problem of air presence in the cardioplegia line. All of the participants answered “no” to the question of whether there was a heat exchanger water leak during the CPB. Forty-four percent of the participants had to change the tube set during CPB. The rate of those who stated that they encountered massive air problems in the arterial line was 22%.

**Fig. 2:**
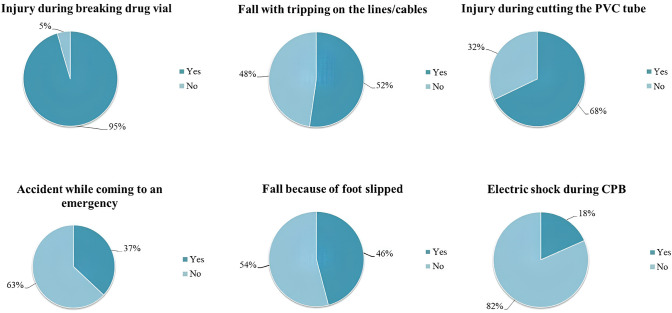
The most common accidents during perfusion pratice. CPB=cardiopulmonary bypass; PVC=polyvinyl chloride.

While 14.7% of the participants stated that they encountered hypothermia problems in the patient at the outlet of CPB due to incorrect measurement of the temperature probe, 1.8% of them encountered malignant hyperthermia. The accidents experienced by perfusionists during their practice were questioned, and the most common accidents are presented in Figure 2.

## DISCUSSION

Considering the number of cases, it can be said that, in general, 83.8% of perfusionists in Turkiye get at least 11 cases in a month^[8]^. Due to these case experiences, it is thought that the perfusionists who participated in the survey provided very important data about perfusion safety and accidents. The first remarkable data from this study is that approximately 1/3 of the perfusionists in Turkiye are female, and 2/3 are male. It can be concluded that Turkish perfusionists are experienced rather than novices, because perfusionists aged 40 years or more correspond to 56.1% of all perfusionists in Turkiye. Most of the perfusionists in our country are highly experienced. In fact, in our study, the rate of perfusionists with ≥ 16 years of experience was 51.8%.

A considerable part of the participants (42.6%, n = 63) had a perfusion master’s degree. It is difficult to say if this rate reflects all perfusionists in Turkiye. However, it can be said that the perfusionists who gave feedback to the surveys are mostly graduates of perfusion associate degree programs.

Turkish perfusionists openly demand a labour contract with a rate of 94.6%. However, all of the perfusionists also demand work/task definition in the affiliated institutions, better description of their limits or responsibilities, recording and archiving the training programs, practice, equipment maintenance, guidelines, and access of these by perfusionists. Of the participants, 85.8% gave the answer of “strongly agree” and 14.2% “agree” to the question that “a checklist should be prepared before the perfusion”, while 58.1% responded as “strongly agree” and 36.5% as “agree” to the question that “a checklist should be prepared after the perfusion”.

One of the interesting results of this study is the willingness of Turkish perfusionists to technology. Of all participants, 55.4% responded as “strongly agree” and 44.6% “agree” to the opinion that an “electronic perfusion database should be used”, and they also statistically showed that they are willing and prone to this issue.

Of the perfusionists, 96.3% established an emergency CPB system. It can be said that the preparation of the device in emergency situations is the process where the risk of making mistakes and the development of perfusion accidents increase the most. On the other hand, nearly 1/3 of perfusionists have the opinion of “undecided” and “disagree” about dry-setup, which can reduce perfusion accidents and increase perfusion safety. This result is thought to be because perfusionists considered the risk of developing a possible infection. The rates of the “undecided” and “disagreed” perfusionists about the use of a pre-bypass filter were 18.9% and 5.4%, respectively. It can be said that there is a need for such studies, since studies on both dry-setup and the use of pre-bypass filters are lacking in our country. We believe that the rates of negative or undecided thoughts will decrease after relevant studies on both issues.

If the perfusionist did not have the chance to use control or install the CPB system in an emergency, reverse connection of the aortic root and intracardiac vent lines may cause a fatal perfusion accident for the patient^[9]^. One of the most important factors that will increase perfusion safety is the use of a one-way valve. Although there was no negative feedback on this issue, the presence of “undecided” perfusionists at a rate of 4.7% suggests that this subject is not discussed very well in perfusionist training.

Another factor that will increase the safety and quality of perfusion is obtaining sufficient and necessary information about the patient by the perfusionist preoperatively^[6]^. In this study, the examination of the patient's file the day before the surgery is accepted by all perfusionists. More than 2/3 of the perfusionists agreed that “the patient should be visited before surgery”. Introducing the perfusionist to the patient, seeing the patient with the naked eye, asking the height and weight verbally, and measuring these values by the perfusionist, if possible, will minimize the wrong applications caused by data errors such as height-weight etc. entered in the file incorrectly while coming to the operating room. By this way, more accurate body surface area and body flow rate values can be calculated, more accurate blood flow will be provided, concomitant metabolic diseases or immune-related problems can be prevented from being missed, mental cooperation of the patients can be observed, and thus, the chance of the perfusionist to compare the patient preoperatively and postoperatively will increase.

The perfusionists want the opportunity to see/monitor the surgical field to ensure perfusion safety in the operating room. Indeed, having control of what happens in the surgical field, which can contribute to safety and quality of perfusion, will reduce errors caused by communication with the surgeon^[10]^. In addition, there will be a chance to intervene earlier, when necessary, in a complication that develops in the surgical field, and thus perfusion safety will be increased. The other suggestions such as the opportunity to work with a double perfusionist who will ensure perfusion safety in the operating room, check of roller pump occlusion settings before the case, routine monthly maintenance of heater-cooler water, measuring ACT and blood gases every half hour, availability of spare gas tubes, and keeping flashlight and hand-crank ready were largely supported by the perfusionists. Although it is generally known by perfusionists what to do in case of malignant hyperthermia in the patient during perfusion, there are few who do not know^[11]^. For this reason, perfusionists should be informed and trained about malignant hyperthermia and its treatment method by institutions. On the other hand, this study shows that Turkish perfusionists generally know what to do when there is a rupture in the arterial line, when the arterial pump module is broken, in case of massive air in the arterial or venous line, in case of dysfunction of the arterial filter, in the presence of massive clot in the venous reservoir, and when the electrical source/battery fails. However, there are some undecided responses about what to do when the heater-cooler device is broken, the oxygen/gas mixer is broken, and the oxygenator needs to be changed, and these perfusionists should also be given the necessary training on these issues.

Regarding the perfusion safety features, the likelihood of death from CPB-related incidents has been declining over the last few decades to 1 in 4446 - 4864 patients, whereas severe injury or death was 1 in 1453 - 3220 patients in the 2000s. Studies on safety and human factors have identified numerous potential risks. It is recommended to objectively report, adequately record, and properly analyze all adverse events related to CPB practice in an efficient and timely manner^[12]^.

A failure mode and effects analysis has identified mechanisms during CPB whereby failing safety equipment or mechanical issues can compromise patient safety. Six different CPB configurations were evaluated. The highest risks across all circuit types were attributed to the embolization of defoamer material, air embolism, spallation, the activation of systemic inflammatory response syndrome, and overpressurization^[13]^. Human factor studies have highlighted several areas for improvement in addition to the mechanical safety of the device, including the organizational culture of safety^[14^,^15]^. Collecting information on adverse events in registries will help prevent such incidents in the future^[10]^. An excellent tool that perfusionists can use for this purpose is the online Perfusion Improvement Reporting System of the Australian and New Zealand College of Perfusionists (https://anzcp.org/pirs-ii/). Perfusion safety can be enhanced by a multitude of measures, such as the use of dedicated safety equipment (*e.g.*, level detectors, bubble detectors, an arterial line filter, pressure transducer, one-way vent valve, backup systems)^[16]^. In a survey published in 2000, 27 safety devices were identified. The authors recommended improvements in coagulation monitoring and incident reporting^[17]^. The question remains whether new developments, such as minimally invasive extracorporeal circulation (MiECC) or surgery without CPB, increase or reduce the safety of perfusion. In a meta-analysis including 134 randomized controlled trials (RCTs), perioperative outcomes were improved by using MiECC or the off-pump technique compared to conventional CPB (for the purpose of these guidelines, ‘conventional CPB’ is defined as CPB not fulfilling the definition of MiECC given in the text and the position paper of the Minimal invasive ExtraCorporeal Technologies international Society)^[18^,^19]^. However, these findings are challenged by large-scale multicentric RCTs^[20^,^21]^.

### Limitations

As of the study period, there were 668 perfusionists registered with the Turkish Perfusionists Society. However, when we consider the perfusionists who are not members of the association, it can be said that the number of perfusionists actively working in Turkiye was around 750 - 800 in 2019. The fact that this limited universe could not be reached is a limitation of this study. Also, other limitations are that the sample consisted of 109 participants for the Perfusion Safety and Accidents survey and 148 participants for the Perfusionists’ Attitudes on Perfusion Safety survey.

## CONCLUSION

The results of this study indicate that Turkish perfusionists are willing to ensure perfusion safety and prevent perfusion accidents. However, they need support in clinical practice regarding perfusion safety. It would be very beneficial to include a course on perfusion safety in academic settings that focuses on this subject in the curricula. In addition, perfusion practice with patient simulators should be integrated into training programs. It is thought that further comprehensive studies on this subject will make very important contributions to both the literature and practical perfusion applications in increasing perfusion safety and reducing perfusion accidents.

## Data Availability

The authors declare that the data supporting the findings of this study are available within the article.

## Artificial Intelligence Usage

The authors declare that no artificial intelligence tool was used in the preparation of this article.
